# Prenatal Edible Bird’s Nest Supplementation Attenuates Offspring Skin Pigmentation via Dual Inhibition of CREB and ERK Signaling to Downregulate MITF-TYR Axis

**DOI:** 10.3390/nu18071083

**Published:** 2026-03-28

**Authors:** Wenrui Zhang, Yijia Zhang, Xinyuan Wang, Yujuan Chen, Liqin Chen, Jie Gao, Yixuan Li, Dongliang Wang, Yanan Sun

**Affiliations:** 1 Key Laboratory of Functional Dairy, Key Laboratory of Precision Nutrition and Food Quality, Department of Nutrition and Health, China Agricultural University, Beijing 100193, China; wrzhang2021@163.com (W.Z.); lxzyj001@163.com (Y.Z.); 15022112227@163.com (X.W.); chenyujuan2000@163.com (Y.C.); liyixuan9735@163.com (Y.L.); 2 Beijing Xiaoxian Dun Biotechnology Co., Ltd., Beijing 100024, China; liqin.chen@xxdun.com (L.C.); shpgj@hebau.edu.cn (J.G.)

**Keywords:** edible bird’s nest, pigmentation, antioxidant, maternal nutrition

## Abstract

Background/Objectives: Edible bird’s nest (EBN) benefits skin, but its transgenerational effects are unknown. This study investigated whether maternal EBN or its key component, sialic acid (SA), could program offspring skin pigmentation and antioxidant capacity. Methods: Pregnant Sprague-Dawley rats were supplemented with EBN or equi-sialic acid SA. Offspring skin brightness (L*, ITA°), melanin content, and key molecular targets (e.g., MITF, TYR, TRP1/2, PMEL, RAB27A, p-CREB, p-ERK, CAT, GCS, MDA) were assessed at postnatal days 0–21. Results: Maternal EBN induced a dose-dependent skin-brightening effect in offspring. High-dose EBN increased skin L* by 10.46% and ITA° by 14.28%, while reducing total melanin by 26.77%. This was mediated by downregulation of the MITF-TYR/TRP axis and its upstream CREB/ERK signaling, suppression of melanosome transport proteins (PMEL, RAB27A), and enhancement of antioxidant defenses (increased CAT/GCS, decreased MDA). SA alone showed similar but weaker effects. Conclusions: This study demonstrates that maternal EBN intake programs offspring skin towards a lighter phenotype and enhanced antioxidant status through multi-faceted regulation of melanogenesis. The superior efficacy of whole EBN over pure SA highlights the value of the intact food matrix, suggesting EBN as a promising functional food for maternal nutrition.

## 1. Introduction

Skin color is primarily determined by the synthesis and distribution of melanin, a pigment produced by melanocytes [1]. Melanin plays a crucial physiological role in photoprotection by absorbing and scattering ultraviolet radiation, thereby shielding the skin from DNA damage [2,3]. However, the overproduction or uneven distribution of melanin leads to hyperpigmentation, which, beyond being a cosmetic concern, can significantly impact psychosocial well-being [4,5]. In more severe cases, persistent hyperpigmentation is associated with dermatological disorders such as melasma, post-inflammatory hyperpigmentation, and mottled pigmentation related to photoaging [6]. These conditions are often challenging to treat and frequently recur, imposing limitations on existing therapeutic strategies including topical depigmenting agents and laser surgery. Notably, skin aging is accompanied by cumulative oxidative stress and cellular senescence, which contribute to pigmentary alterations and dysregulated melanogenesis [6]. Accordingly, the pathogenesis of these pigmentary disorders is complex and considered multifactorial, involving mechanisms such as oxidative stress, chronic low-grade inflammation, and dysregulated melanocyte–keratinocyte interactions.

Edible bird’s nest (EBN), the edible, glutinous salivary cement of Southeast Asian swiftlets (primarily of the genera Aerodramus or Collocalia), is a traditionally consumed nutrient-rich functional food ingested for its purported skin and health benefits, recognized in maternal and infant dietary practices [7,8,9,10]. Modern science has progressively revealed that its biological activities arise from a complex mixture of bioactive peptides, glycoproteins (including several forms of bound sialic acids as major components), growth factor-like substances, and antioxidants within its intricate matrix structure, this multifactorial composition underpins its proposed holistic health effects, distinguishing it from single-component supplements [11,12,13,14]. Numerous consumer studies across East and Southeast Asian countries have validated its sustained popularity; however, given species variation and harvesting regulation constraints, standardized preparations for research purposes are required [15,16,17]. Modern studies show that EBN exhibits antioxidant, anti-inflammatory, photo-protective and pro-collagen effects in in vitro, animal, and human models [18,19,20]. Pepsin-digested EBN and EBN-derived peptides reduce melanin content and tyrosinase activity in B16 melanoma cells and zebrafish and downregulate MITF-TYR-TRP1/2 axis expression. Clinical trials suggest oral EBN extracts improve skin wrinkles, elasticity, and brightness in women [21].

Epidermal pigmentation is governed by melanogenesis within melanosomes of melanocytes and by subsequent transfer of mature melanosomes to keratinocytes [22,23]. The rate-limiting enzyme tyrosinase (TYR) catalyzes hydroxylation of L-tyrosine to L-DOPA and its oxidation to DOPA-quinone, while tyrosinase-related proteins 1 and 2 (TRP1/TYRP1 and TRP2/DCT) support downstream steps leading to eumelanin and pheomelanin synthesis. These enzymes are transcriptionally regulated by microphthalmia-associated transcription factor (MITF), the master regulator of melanocyte development and function [24,25].

MITF is coordinately regulated by several upstream signaling pathways, most notably the melanocortin-1 receptor (MC1R)/cAMP/PKA/CREB axis and the MAPK/ERK pathway [26,27]. Binding of α-MSH or ACTH to MC1R increases intracellular cAMP, activates PKA, and induces phosphorylation of CREB at Ser133. Phosphorylated CREB (p-CREB) then enhances MITF gene transcription [28]. Concurrently, the activity of MITF is also modulated by the MAPK/ERK pathway. Inhibition or downregulation of ERK1/2 signaling, reflected by reduced phosphorylation at its activation loop residues, has been consistently associated with decreased MITF expression and a subsequent reduction in melanogenesis in various experimental models [29,30]. Thus, suppression of either the p-CREB/CREB or the p-ERK/ERK signaling axis can converge to downregulate MITF and inhibit melanin production. Consequently, many depigmenting agents function by targeting and attenuating these critical signaling cascades [31].

Visible pigmentation further depends on melanosome transport within melanocytes and their transfer to keratinocytes [23,32]. Rab27A, melanophagin, and myosin Va form a motor complex essential for peripheral melanosome transport, while Melanoma gp100 is critical for melanosome maturation [32]. On the keratinocyte side, protease-activated receptor-2 (PAR-2) promotes phagocytic uptake of melanosomes; inhibiting PAR-2 leads to skin lightening [33].

Reactive oxygen species (ROS) generated by ultraviolet (UV) radiation, pollution, and endogenous metabolism are key drivers of oxidative stress in skin, contributing directly to cellular damage and stimulating melanogenesis, which leads to hyperpigmentation. A critical defense against this damage is the antioxidant enzyme system, with catalase (CAT) playing a central role in decomposing hydrogen peroxide, a major ROS [34]. The balance between ROS production and scavenging by antioxidants like CAT determines the degree of oxidative stress, which can be assessed by the levels of lipid peroxidation products such as malondialdehyde (MDA). Furthermore, the synthesis of intracellular glutathione, a master antioxidant, is regulated by glutamate–cysteine ligase (GCS), highlighting its importance in the skin’s antioxidant network. Notably, oxidative stress can activate melanogenic signaling, while enhancing the endogenous antioxidant capacity through upregulating enzymes like CAT and GCS has been shown to suppress melanin production [35,36]. Therefore, strategies that simultaneously enhance key antioxidant defenses (such as CAT and GCS) and reduce oxidative damage (such as MDA) represent a promising intervention mechanism for attenuating skin melanogenesis [37]. Importantly, evidence from the Developmental Origins of Health And Disease (DOHaD) field indicates that redox status in early life, including the fetal period, acts as a critical environmental signal that can shape long-term tissue function in organs such as the liver, nervous system, and skin. Consistently, maternal antioxidant status has been reported to influence offspring outcomes by modulating the developmental programming of antioxidant enzyme systems and oxidative damage indices (e.g., CAT/GSH-related pathways and MDA), with effects that may persist postnatally [38].

The Developmental Origins of Health and Disease (DOHaD) framework posits that environmental exposures during critical developmental windows—particularly maternal nutrition—can durably shape offspring phenotypes via long-lasting changes in regulatory systems, including epigenetic, endocrine, immune, and metabolic pathways [39,40]. In this context, redox balance during embryonic and fetal development is increasingly recognized as an integral component of developmental programming, because developing tissues have limited antioxidant buffering capacity and are sensitive to oxidative cues that may influence postnatal tissue function [41]. Notably, melanocytes arise from neural crest-derived progenitors and undergo specification, migration, and differentiation during development, suggesting that melanocyte lineage regulation and pigmentation-related networks may be susceptible to early-life environmental signals [42]. Together with the established mechanistic links between oxidative stress and melanogenesis in pigmentary disorders [43], these concepts support the biological plausibility that maternal intake of bioactive, antioxidant-rich foods such as EBN during gestation could influence offspring pigmentation and cutaneous antioxidant defenses beyond birth.

Given the brightening and photo-protective actions of EBN in adult models, the central roles of MITF-centered melanogenic pathways, melanosome transport, and oxidative stress in pigmentation [21,44], we hypothesized that maternal EBN consumption during pregnancy would program offspring skin toward a lighter, less melanogenic, and more antioxidant-competent state. Therefore, we established a rat model in which dams received vehicle, SA, low-dose EBN or high-dose EBN during gestation and comprehensively evaluated pigmentary and redox endpoints in offspring dorsal skin from birth to weaning.

## 2. Materials and Methods

### 2.1. Edible Bird’s Nest (EBN) Preparation and Dosing Solution

The cleaned, dried, and raw edible bird’s nest (EBN) material used in this study was imported from Indonesia. To prepare the oral dosing solution for pregnant dams, EBN was processed as follows: briefly, 5 g of dried EBN was dissolved in 95 mL of pure water, and the mixture was boiled at 95 °C for 15 min to obtain a homogeneous solution. This yielded a 5% (*w*/*v*) aqueous EBN extract, which was used as the administration solution after cooling to room temperature. The solution was prepared fresh daily throughout the gestation period to ensure stability and consistency.

### 2.2. Animals and Ethics

Female (*n* = 24) and male (*n* = 12) specific-pathogen-free Sprague-Dawley rats (10 weeks old) were obtained from Sipeifu (Beijing, China). Animals were maintained under controlled conditions (22 ± 2 °C; 50 ± 10% humidity; 12/12 h light/dark cycle) with ad libitum access to standard chow and water. All procedures were approved by the Institutional Animal Care and Use Committee of Pony Testing International Group (Approval No. PONY-2023-FL-27) and conducted in accordance with relevant guidelines.

### 2.3. Experimental Design and Treatment Groups

After one week of acclimatization, rats were randomly allocated into four groups (6 females + 3 males per group):

Control: Vehicle (CK): distilled water or PBS; 2 mL/day, oral gavage; continuous gastric lavage for 21 days;

Sialic acid (SA): Sialic acid standard at 0.045 g/kg body weight; continuous gastric lavage for 21 days;

Low-dose EBN (LEBN): Homogenate of fresh-steamed EBN at 4.5 g/kg body weight; continuous gastric lavage for 21 days;

High-dose EBN (HEBN): Homogenate of fresh-steamed EBN at 9.0 g/kg body weight, continuous gastric lavage for 21 days.

Doses were selected based on preliminary tolerability and previous EBN supplementation studies [45]. Dosing Rationale: The sialic acid (SA) group received 0.045 g SA/kg body weight. The EBN low- and high-dose groups received 4.5 and 9.0 g EBN/kg, respectively. This dosing scheme was designed to be equi-sialic acid: based on our analysis and the supplier’s certificate, the EBN used contains approximately 0.5% (*w/w*) sialic acid, primarily existing in its glycoprotein-bound form as N-acetylneuraminic acid (Neu5Ac) residues, rather than as free monomers. Thus, 9.0 g/kg EBN delivers roughly 0.045 g SA/kg, matching the dose in the pure SA group. This design allows for the direct comparison of the whole EBN matrix effect against its isolated key component at an equivalent sialic acid dose.

### 2.4. Mating and Gestational Administration

For mating, two female Sprague-Dawley rats were housed with one male per cage. The presence of a vaginal plug was designated as gestational day (GD) 0. From GD0 until parturition, pregnant dams received their assigned treatments once daily via oral gavage, with a volume of 2 mL per administration. Treatment ceased on the day of delivery. Litter size and pup viability were recorded at birth.

### 2.5. Offspring Sampling and Skin Collection

Offspring were evaluated at postnatal days (P) 0, 7, 14, and 21. At each time point, a subset of pups from each litter was anesthetized and euthanized humanely. The dorsal skin was shaved and cleaned. Skin colorimetric parameters were measured in vivo immediately thereafter. Subsequently, the skin was surgically excised. One portion of the skin sample was fixed in 4% paraformaldehyde for 24 h for subsequent paraffin embedding, while another portion was snap-frozen in liquid nitrogen and stored at −80 °C for biochemical, RNA, and protein analyses. To minimize potential litter effects, no more than 2–3 pups per sex from each litter were used at any given time point.

### 2.6. Skin Color Measurements

Skin color on the shaved dorsal area was measured using a handheld chromameter (ColorMeter Pro D/8, Hangzhou CHNSpec Technology Co., Ltd., Hangzhou, China). The instrument was calibrated using a standard white and black tile prior to measurements. For newborn pups (P0 and P7), skin color was measured directly on the shaved dorsal area as described above. At later time points (P14 and P21), offspring developed a visible coat. Prior to colorimetry, the dorsal hair was carefully removed using a gentle animal clipper followed by a depilatory cream (Veet^®^, Slough, UK) applied for no more than 2 min and thoroughly rinsed with warm water. The skin was then gently patted dry and allowed to rest for at least 30 min to allow any transient erythema (skin redness due to hair removal) to subside, ensuring that the measurement reflected baseline pigmentation rather than an inflammatory response. For all measurements, the chromameter probe was placed perpendicular to and in gentle, consistent contact with the skin surface. Three consecutive measurements were taken at the mid-dorsal region, and the average values of the L* (lightness), a* (red-green), and b* (yellow-blue) parameters were recorded for analysis. The Individual Typology Angle (ITA°) was calculated using the formula:

$$\mathrm{ITA}{}^{\circ}\;=\;\arctan(\frac{L^{*}-50}{b^{*}})\times \frac{180}{\Pi }$$

Higher L* and ITA° indicate lighter skin.

### 2.7. Quantification of Melanin Content, Tyrosinase Activity, and MITF Protein

At P0, P7, P14, and P21, frozen skin tissues were homogenized in ice-cold phosphate-buffered saline or specific assay buffers provided with the commercial kits. The concentrations of total melanin, eumelanin, and pheomelanin were quantified using species-specific ELISA kits: Total Melanin: Rat Melanin ELISA Kit (Cat# YJ-68752, Shanghai Yuanjie Bio-Technology Co., Ltd., Shanghai, China); Eumelanin: Rat Eumelanin ELISA Kit (Cat# YJ-44210, Shanghai Yuanjie Bio-Technology Co., Ltd., Shanghai, China); Pheomelanin: Rat Pheomelanin ELISA Kit (Cat# YJ-77023, Shanghai Yuanjie Bio-Technology Co., Ltd., Shanghai, China) according to the manufacturer’s instructions; a corresponding pheomelanin ELISA kit was used according to the manufacturer’s protocol. The reliability of the assays for our sample matrix was confirmed by satisfactory standard curve linearity (R^2^ > 0.99), spike-and-recovery tests (mean recovery: 95–102%), and acceptable precision (CV < 10%). Tyrosinase Activity Assay Kit (Cat# AKAM010M, Beijing Box Shenggong Technology Co., Ltd., Beijing, China) and Rat microphthalmia-associated transcription factor (MITF) ELISA Kit (Cat# RX2D392766, Quanzhou Ruixin Biotechnology Co., Ltd., Quanzhou, China), respectively. All assays were performed strictly according to the manufacturers’ instructions, and absorbance was read using a microplate reader. For histological assessment of melanin deposition, paraffin-embedded sections were stained using the Masson–Fontana silver stain method.

### 2.8. Immunohistochemistry (IHC) and Immunofluorescence (IF)

Paraffin-embedded skin sections (4–5 μm thickness) were deparaffinized, rehydrated, and subjected to antigen retrieval. For IHC, sections were incubated with primary antibodies against tyrosinase (Anti-tyrosinase antibody [TYR/2024R], Rabbit Recombinant Monoclonal, Cat# ab236495, Abcam, Cambridge, UK) or MITF (MITF Rabbit polyclonal antibody, Cat# ab12039, Abcam) overnight at 4 °C. After washing, sections were incubated with an HRP-conjugated secondary antibody, and color was developed using a 3,3′-diaminobenzidine (DAB) chromogen kit. Sections were counterstained with haematoxylin. For IF, sections were incubated with primary antibodies against PMEL/gp100 (Anti-Melanoma gp100 antibody, Cat# ab27435, Abcam) and RAB27A (Anti-RAB27A + RAB27B antibody, Cat# ab192673, Abcam) overnight at 4 °C. After washing, appropriate fluorescent secondary antibodies (e.g., Alexa Fluor 488-conjugated anti-rabbit and 594-conjugated anti-mouse) were applied, and nuclei were counterstained with DAPI.

Immunohistochemistry images were acquired using a Nikon Eclipse ci upright microscope equipped with a Nikon DS-Fi2 digital camera system (Nikon Instruments Inc., Melville, NY, USA). The microscope was controlled by NIS-Elements BR software (Ver. 4.30.00, 64-bit). For IF, specific filter sets were used for different channels: DAPI (Ex: 340–380 nm, Em: 435–485 nm), FITC/Alexa Fluor 488 (Ex: 465–495 nm, Em: 515–555 nm), and TRITC/Alexa Fluor 594 (G-2A filter set, Ex: 510–560 nm, Em: 590 nm).

Image acquisition and semi-quantification were performed under consistent settings. For each section, three to five non-overlapping fields of view were randomly captured at 40× or 200× magnification. The staining intensity and positive area for IHC, and the mean fluorescence intensity (MFI) for IF, were analyzed using ImageJ software (version 1.8.0; National Institutes of Health, Bethesda, MD, USA).

### 2.9. Quantitative Real-Time PCR (qPCR)

Total RNA was extracted from frozen dorsal skin using TRIzol Reagent (Cat# 15596026, Invitrogen, Waltham, MA, USA). RNA concentration and purity were assessed spectrophotometrically. Complementary DNA (cDNA) was synthesized from 1 μg of total RNA using a reverse transcription kit (Prime Script RT Master Mix, Takara, Shiga, Japan). Quantitative PCR was performed using SYBR Green Master Mix (Cat# 4309155, Applied Biosystems, Waltham, MA, USA) on a Quant Studio real-time PCR system (Applied Biosystems). The primer sequences for target genes are listed in Supplementary Table S1. The genes analyzed included: melanogenesis-related (TYR, TRP1, TRP2, MITF, MC1R, POMC); and antioxidant-related (CAT, GCS). GAPDH or β-actin served as the internal reference genes. The relative mRNA expression levels were calculated using the 2^−ΔΔCt^ method.

### 2.10. Assessment of Oxidative Stress and Antioxidant Indices

Skin homogenates were prepared in cold buffer. Malondialdehyde (MDA) levels, a marker of lipid peroxidation, were measured using a Rat MDA ELISA Kit (Cat# YJ-23653, Shanghai Yuanjie Bio-Technology Co., Ltd., Shanghai, China). Catalase (CAT) activity was assessed using a Rat CAT ELISA Kit (Cat# AKAO003-2M, Beijing Box Shenggong Technology Co., Ltd., Beijing, China). Absorbance was measured using a microplate reader, and results were normalized to the total protein concentration of the homogenate.

### 2.11. Western Blot Analysis

Total protein was extracted from P21 skin tissues using RIPA lysis buffer supplemented with protease and phosphatase inhibitor cocktails. Protein concentration was determined using the Pierce BCA Protein Assay Kit (Cat# 23225, Thermo Fisher Scientific, Waltham, MA, USA). Equal amounts of protein (30 μg) were separated by 10% SDS-polyacrylamide gel electrophoresis (SDS-PAGE) and electrophoretically transferred onto PVDF membranes.

The membranes were then blocked with 5% non-fat milk in Tris-buffered saline containing 0.1% Tween-20 (TBST) for 1 h at room temperature. Following blocking, the membranes were incubated overnight at 4 °C with the following primary antibodies from Abcam: anti-CREB (Cat# ab32515, 1:1000 dilution), anti-phospho-CREB (Ser133) (Cat# ab32096, 1:1000 dilution), anti-ERK1/2 (Cat# ab17942, 1:1000 dilution), and anti-phospho-ERK1/2 (Thr202/Tyr204) (Cat# ab128159, 1:1000 dilution). After three 10 min washes with TBST, the membranes were incubated with appropriate HRP-conjugated secondary antibodies for 1 h at room temperature. Protein bands were visualized using an enhanced chemiluminescence (ECL) detection substrate (Pierce™ ECL Western Blotting Substrate, Cat# 32106, Thermo Fisher Scientific) and captured with a chemiluminescence imaging system.

For data analysis, band intensities were quantified using ImageJ software (National Institutes of Health). GAPDH (anti-GAPDH, Cat#ab9485, abcam, 1:5000 dilution) was used as a loading control to correct for minor variations in total protein loading. To assess the activation status of signaling pathways, the following normalization procedure was performed: The intensity of each phosphorylated protein band (p-CREB or p-ERK1/2) was first normalized to the intensity of its corresponding total protein band (total CREB or total ERK1/2, respectively) from the same membrane to obtain the p/total ratio. Subsequently, this p/total ratio was further normalized to the GAPDH level from the same lane to account for differences in total protein loaded per lane. These final normalized values were used for statistical analysis between treatment groups.

### 2.12. Statistical Analysis

All data are presented as the mean ± standard deviation (SD). The normality of all data sets was confirmed prior to analysis using the Shapiro–Wilk test. To account for the litter effect and avoid pseudoreplication, which is critical in developmental exposure studies, the litter was treated as the independent experimental unit. Specifically, at each postnatal time point (P0, P7, P14, P21), 1–2 pups per sex were randomly sampled from each litter for measurement. For each outcome variable, the values from the sampled pups within a given litter and time point were averaged to generate a single litter-mean value. These litter-means, rather than individual pup data, were used in all subsequent statistical analyses. Given the primary focus of this study on establishing the overall transgenerational effect of maternal EBN intake and considering the practical challenges in reliably sexing neonatal pups for all analyses, data from male and female offspring were pooled for the primary statistical comparisons. Preliminary assessment indicated no overt sex-specific trends in the key outcome measures (skin L* value).

Since data were collected from independent animal cohorts at each time point (P0, P7, P14, P21), a repeated-measures model was not applicable. For comparisons among the four treatment groups (control, SA, L-EBN, H-EBN) at each time point, one-way analysis of variance (ANOVA) was performed, followed by Tukey’s post hoc test for multiple comparisons. In selected analyses involving two groups, Student’s *t*-test was used. A *p*-value of less than 0.05 was considered statistically significant. All statistical analyses were conducted using GraphPad Prism software (version 8.0.2, GraphPad Software, San Diego, CA, USA).

## 3. Results

### 3.1. Maternal EBN Supplementation Increases Offspring Skin Lightness in a Dose- and Age-Dependent Manner

**Figure 1:**
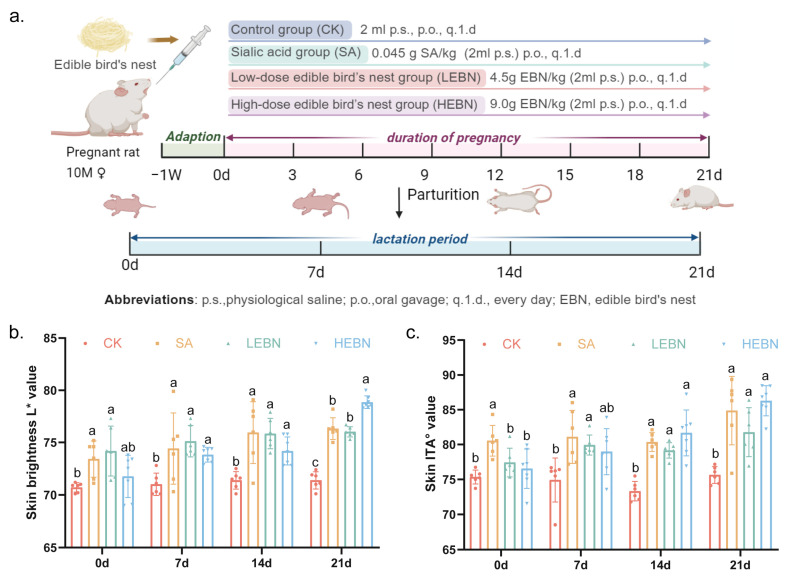
Maternal EBN supplementation protocol and effects on offspring skin color. (**a**) Schematic diagram of experimental design. Female Sprague-Dawley rats received vehicle (control), sialic acid (SA, 0.045 g SA/kg/d), low-dose edible bird’s nest (LEBN, 4.5 g of EBN/kg/d) or high-dose edible bird’s nest (HEBN, 9.0 g of EBN/kg/d) by oral gavage throughout gestation. Offspring dorsal skin was collected at postnatal days 0 (P0), 7 (P7), 14 (P14), and 21 (P21). (**b**) Dorsal skin lightness (L* value) of offspring at P0, P7, P14, and P21 in the four maternal groups. (**c**) Individual typology angle (ITA°) values of dorsal skin at P0, P7, P14, and P21. Data points represent litter means. Data from male and female offspring were pooled for the primary analysis of the maternal treatment effect. Data are expressed as mean ± SD (*n* = 5 per group). Different letters above bars indicate significant differences among groups (*p* < 0.05).

All offspring phenotypic data presented in this and subsequent sections are derived from litter means, which served as the independent statistical units for analysis, as detailed in Section 2.12. The experimental design is illustrated in Figure 1a. At P0, both EBN groups exhibited significantly higher L* and ITA° values than the control and SA groups (*p* < 0.05), with H-EBN showing the highest values (Figure 1b,c). This trend became more pronounced at P7, P14, and P21. At all points, L* and ITA° followed a hierarchical order: HEBN > LEBN > SA > control group (HEBN vs. control group, *p* < 0.05; LEBN significantly higher than the control group at most time points, *p* < 0.05). Notably, by day 21, maternal intake of high-dose bird’s nest significantly increased offspring skin brightness (L* value) and individual type angle (ITA°) compared to the control group (brightness increased by 10.46%, ITA° increased by 14.28%). Maternal EBN intake thus increased offspring skin lightness in a manner that was both dose-dependent and progressively more evident with advancing postnatal age.

### 3.2. EBN Reduces Total Melanin, Eumelanin, and Pheomelanin Content

**Figure 2:**
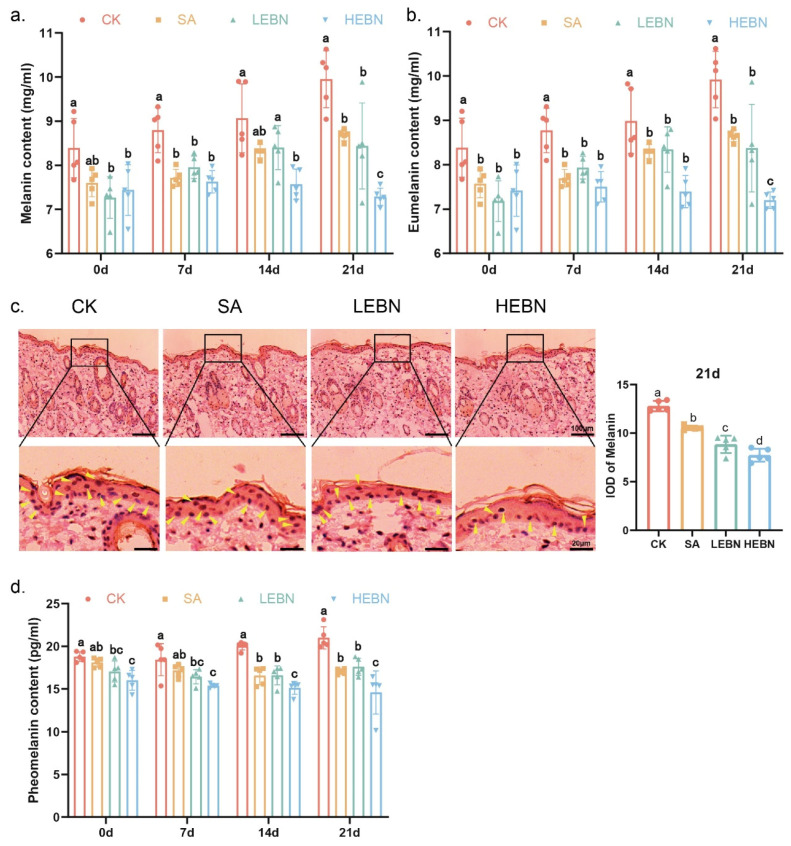
Maternal EBN supplementation reduces melanin, eumelanin, and pheomelanin in offspring skin. (**a**) Total melanin content in dorsal skin at P0, P7, P14, and P21 in control, SA, LEBN, and HEBN groups. (**b**) Eumelanin content in dorsal skin at P0, P7, P14, and P21. (**c**) Representative Masson–Fontana staining images of dorsal skin sections at each time point showing melanin deposition in the basal epidermis (arrows indicate melanin granules). Scale bar as indicated. (**d**) Pheomelanin content in dorsal skin at P0, P7, P14, and P21. Data points represent litter means. Data from male and female offspring were pooled for the primary analysis of the maternal treatment effect. Data are expressed as mean ± SD (*n* = 5 per group). Different letters above bars indicate significant differences among groups (*p* < 0.05).

At all assessment time points, total melanin content in dorsal skin was significantly lower across all treatment groups compared to the control group, with HEBN exhibiting the greatest reduction (*p* < 0.05; Figure 2a). Masson–Fontana staining corroborated these findings, revealing progressively diminished melanin deposition and reduced intensity within the basal epidermis of EBN-treated offspring, most markedly evident in HEBN (Figure 2c). From P0 to P21, eumelanin and pheomelanin contents also decreased in a dose-dependent manner (HEBN < LEBN < SA < control) (Figure 2b,d). By day 21, compared to the control group, the high-dose bird’s nest group exhibited a 26.77% reduction in total melanin, a 27.41% decrease in eumelanin, and a 30.49% reduction in pheomelanin. The parallel reduction in eumelanin and pheomelanin indicates an overall inhibition of melanin formation rather than a selective shift in melanin subtypes. Thus, prenatal EBN intervention dose-dependently reduced the two primary forms of melanin, providing a biochemical and histomorphological basis for the observed skin lightening.

### 3.3. EBN Inhibits Tyrosinase Activity and Melanogenic Gene Expression

**Figure 3:**
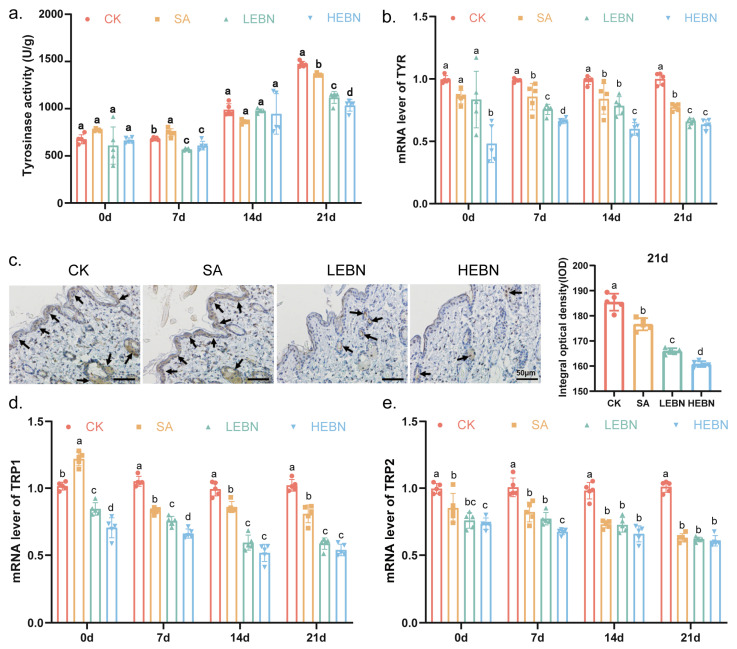
Maternal EBN supplementation decreases tyrosinase activity and expression of melanogenic enzymes in offspring skin. (**a**) Tyrosinase activity in dorsal skin homogenates at P0, P7, P14, and P21. (**b**) Relative TYR mRNA expression in dorsal skin at P0, P7, P14, and P21. (**c**) Immunohistochemical staining images of tyrosinase (TYR) in dorsal skin sections from offspring of each maternal group at P21. The arrows indicate representative TYR-positive cells (brown staining). Statistical results of the positive signals are shown alongside. (**d**) Relative TRP1 mRNA expression in dorsal skin at P0, P7, P14, and P21. (**e**) Relative TRP2 mRNA expression in dorsal skin at P0, P7, P14, and P21. Gene expression was normalized to GAPDH using the 2^−ΔΔCt^ method. Data points represent litter means. Data from male and female offspring were pooled for the primary analysis of the maternal treatment effect. Data are expressed as mean ± SD (*n* = 5 per group). Different letters above bars indicate significant differences among groups (*p* < 0.05).

Compared with the control group, tyrosinase activity in skin homogenates from the SA, LEBN, and HEBN groups was significantly reduced at all time points (*p* < 0.05; Figure 3a). By day 21, TYR activity in the high-dose bird’s nest group decreased by 29.77% compared to the control group. Immunohistochemistry revealed markedly reduced TYR staining intensity in the basal layer of offspring treated with EBN, particularly in the HEBN group (Figure 3b). By day 21, TYR staining intensity in the high-dose bird’s nest group decreased by 13.28% relative to the control group. At the transcriptional level, TYR, TRP1, and TRP2 mRNA expression was significantly downregulated in all treatment groups compared to the control, with the strongest suppression observed in the HEBN group (Figure 3b,d,e). By P21, HEBN pups exhibited the lowest expression of all three melanogenesis enzymes. Compared to the control group, the high-dose bird’s nest group showed a 36.49% reduction in TYR gene expression, a 47.31% decrease in TRP1 gene expression, and a 39.99% decline in TRP2 gene expression. Together, these data demonstrate that maternal EBN supplementation suppresses melanogenesis at the level of both enzyme activity and gene expression. Data for qPCR and ELISA analyses were derived from one randomly selected offspring per litter to ensure that each data point (*n* = 5 per group) represented an independent biological replicate (i.e., a distinct litter).

### 3.4. Maternal EBN Supplementation Downregulates the Central Melanogenic Transcription Factor MITF in Offspring Skin

**Figure 4:**
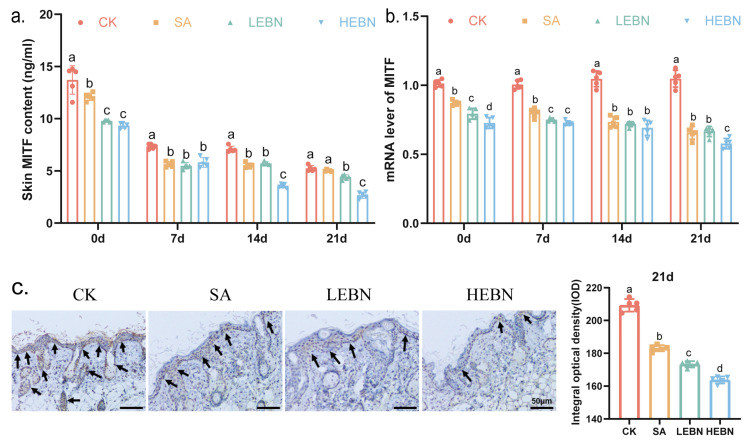
Maternal EBN supplementation suppresses MITF expression in offspring skin. (**a**) MITF protein levels in dorsal skin at P0, P7, P14, and P21 measured by ELISA. (**b**) Relative MITF mRNA expression in dorsal skin at P0, P7, P14, and P21. (**c**) Representative immunohistochemical staining for MITF in dorsal skin sections at P21 in control, SA, LEBN, and HEBN groups. Arrows indicate MITF-positive nuclei (brown staining). Data points represent litter means. Data from male and female offspring were pooled for the primary analysis of the maternal treatment effect. Data are expressed as mean ± SD (*n* = 5 per group). Different letters above bars indicate significant differences among groups (*p* < 0.05).

Next, we examined MITF, a key regulator of melanogenesis. ELISA and immunohistochemical analyses of P21 skin tissue revealed that the HEBN group exhibited the lowest MITF protein levels, followed by the low-dose group and SA group (Figure 4a). By day 21, MITF activity in the high-dose bird’s nest group decreased by 49.09% compared to the control group (Figure 4b,c). Immunohistochemistry confirmed reduced MITF staining in the basal layer following EBN treatment. By day 21, MITF staining intensity decreased by 21.75% in the HEBN group compared to the control group (Figure 4b). MITF mRNA expression was also diminished in the treatment groups, particularly at P14 and P21. By day 21, high-dose bird’s nest group MITF gene expression decreased by 44.9% compared to the control group (Figure 4c). Therefore, the downregulation of MITF at the protein and mRNA levels provides a direct mechanistic link to the observed suppression of its downstream target enzymes, TYR, TRP1, and TRP2.

### 3.5. Maternal EBN Supplementation Inhibits Offspring Melanogenesis by Concurrently Suppressing CREB and ERK Signaling

**Figure 5:**
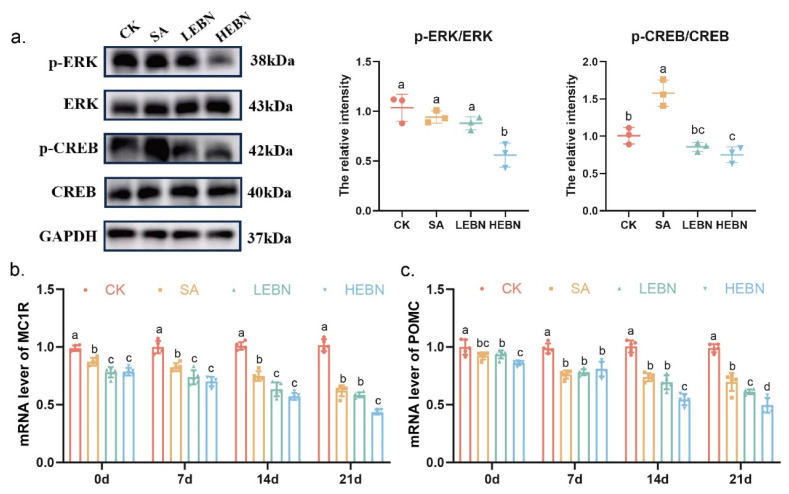
Maternal EBN supplementation concurrently inhibits the phosphorylation of CREB and ERK and downregulates upstream melanocortin signaling in offspring skin. (**a**) Parallel regulation of MITF by CREB and ERK pathways. Representative Western blots and densitometric analysis of phospho-CREB (p-CREB), total CREB, phospho-ERK1/2 (p-ERK1/2), and total ERK1/2 in dorsal skin at P21. Bar graphs show the p-CREB/CREB and p-ERK/ERK ratios (*n* = 3 independent biological replicates per group, each replicate consisting of skin tissue pooled from offspring within one litter). (**b**,**c**) Upstream gene expression. Relative mRNA expression levels of (**b**) MC1R and (**c**) POMC in dorsal skin at P0, P7, P14, and P21. Data are expressed as mean ± SD (*n* = 5 per group). Different letters above bars indicate significant differences among groups (*p* < 0.05).

To elucidate the upstream mechanisms by which maternal EBN supplementation inhibits melanogenesis in offspring, we examined key regulatory nodes. qPCR analysis revealed that EBN treatment significantly downregulated the expression of MC1R and POMC, which encode critical upstream components of melanogenic signaling. By postnatal day 21, the high-dose EBN group exhibited reductions of 57.10% in Mc1r mRNA and 49.98% in POMC mRNA compared to the control group (Figure 5b,c).

We further investigated the activity of two parallel signaling cascades converging on MITF: the cAMP/PKA-CREB pathway and the MAPK/ERK pathway. Western blot analysis demonstrated that maternal EBN supplementation significantly decreased the phosphorylation states of both CREB and ERK1/2 in offspring skin. Specifically, the p-CREB/CREB ratio was reduced by 25.50%, and the p-ERK/ERK ratio was reduced by 47.67% in the high-dose EBN group compared to controls at P21 (Figure 5a). This coordinated downregulation of both CREB and ERK phosphorylation indicates a broad suppression of the melanogenic signaling network. Importantly, the observed reduction in ERK1/2 phosphorylation should be interpreted within the context-dependent role of ERK in melanogenesis. While sustained ERK activation can promote MITF degradation in certain contexts (e.g., stress responses), ERK is also an integral component of pro-melanogenic cascades initiated by receptors like c-Kit. The attenuation of ERK signaling observed here likely reflects the suppression of such a pro-melanogenic signal flux, contributing to the overall downregulation of the MITF-TYR axis. Given that Western blotting necessitated pooling skin tissue from P21 offspring within each litter to obtain sufficient protein, each data point (*n* = 3 per group) represents an independent biological replicate derived from a separate litter (rather than individual pups). These results point to a mechanism whereby maternal EBN intake attenuates pigmentation by suppressing the upstream MC1R/POMC axis and concurrently inhibiting the phosphorylation and activation of the key MITF regulators, CREB and ERK.

### 3.6. EBN Attenuates Melanosome Maturation and Transfer

**Figure 6:**
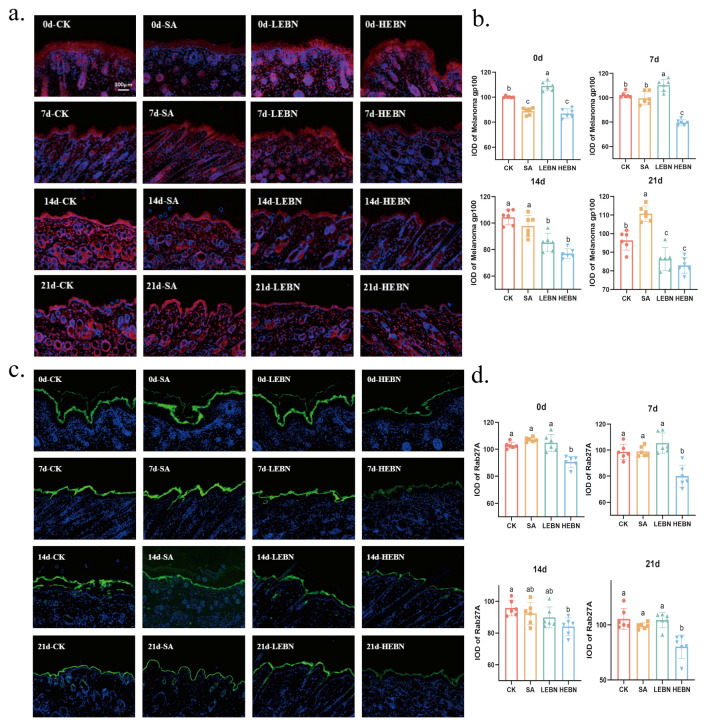
Maternal supplementation with EBN inhibits melanin granule maturation and transport in offspring skin. (**a**) Representative immunofluorescence images of melanoma gp100 in dorsal skin from P0, P7, P14, and P21 groups. (Red signal). (**b**) Quantitative analysis of average fluorescence intensity for melanoma gp100. (**c**) Representative immunofluorescence staining images of Rab27A in dorsal skin at P0, P7, P14, and P21 time points. (Green signal). (**d**) Quantitative analysis of mean fluorescence intensity for Rab27A. Cell nuclei counterstained with DAPI (blue signal). Scale bar as shown. Data are expressed as mean ± SD (*n* = 5 per group). Data points represent litter means. Data from male and female offspring were pooled for the primary analysis of the maternal treatment effect. Different letters on the bar indicate significant differences between groups (*p* < 0.05).

Immunofluorescence staining revealed that at all time points, the fluorescence intensities of melanin body maturation Melanoma gp100 and transport protein RAB27A in both the LEBN and HEBN groups were significantly lower than those in the control and SA groups. By day 21, compared with the control group, the fluorescence intensity of Melanoma gp100 in the high-dose bird’s nest group decreased by 13.94%, while RAB27A fluorescence intensity decreased by 23.93% (*p* < 0.05; Figure 6a,b). H-EBN signals were weakest, indicating reduced Melanoma gp100 and RAB27A. Thus, in addition to inhibiting melanin synthesis, maternal EBN intake appears to compromise melanosome maturation and intracellular transport. A similar sampling strategy was applied, with one representative section from one pup per litter used for analysis, providing *n* = 5 biological replicates (litters) per group.

### 3.7. EBN Enhances Antioxidant Capacity and Reduces Oxidative Stress in Offspring Skin

**Figure 7:**
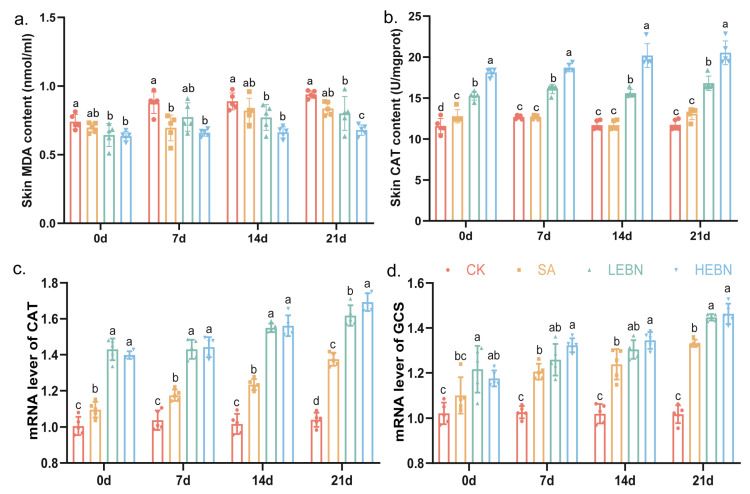
Maternal EBN supplementation enhances antioxidant defense and reduces oxidative stress in offspring skin. (**a**) Malondialdehyde (MDA) content in dorsal skin at P21 in control, SA, L-EBN and H-EBN groups. (**b**) Catalase (CAT) activity in dorsal skin at P21 in control, SA, L-EBN, and H-EBN groups. (**c**) Relative mRNA expression of CAT in dorsal skin at P0, P7, P14, and P21. (**d**) Relative mRNA expression of GCS in dorsal skin at P0, P7, P14, and P21. Gene expression was normalized to GAPDH or β-actin using the 2^−ΔΔCt^ method. Data points represent litter means. Data from male and female offspring were pooled for the primary analysis of the maternal treatment effect. Data are presented as mean ± SD. (*n* = 5 per group). Different letters above bars indicate significant differences among groups (*p* < 0.05).

At P21, compared with the control group, MDA levels were significantly reduced in the SA, LEBN, and HEBN groups, with the lowest levels observed in the HEBN group. By day 21, the high-dose bird’s nest group exhibited a 27.60% reduction in MDA levels relative to the control group (*p* < 0.05; Figure 7a). In contrast, CAT activity was markedly higher in the EBN groups than in the control group, particularly in the H-EBN group. By day 21, CAT activity in the high-dose bird’s nest group increased by 74.95% compared to the control group (*p* < 0.05; Figure 7b). qPCR analysis revealed significantly upregulated CAT and GCS mRNA levels in the EBN group, most markedly in the H-EBN group. At 21 days, compared with the control group, the high-dose bird’s nest group exhibited a 62.85% upregulation in CAT gene expression and a 43.84% increase in GCS gene expression (*p* < 0.05; Figure 7c,d). These data show that maternal EBN supplementation enhances the offspring skin’s antioxidant defense system while reducing oxidative damage, creating a tissue microenvironment less conducive to melanogenesis.

## 4. Discussion

This study demonstrates that maternal supplementation with edible bird’s nest (EBN) during pregnancy effectively regulates skin pigmentation in offspring and enhances their antioxidant capacity. Skin from EBN-treated offspring exhibited a distinct brightening phenotype, characterized by increased L* and ITA° values, decreased levels of total melanin, eumelanin, and pheomelanin, alongside suppressed tyrosinase activity and related gene expression. Further mechanistic analysis revealed that EBN downregulates MITF and its upstream MC1R/cAMP-CREB and ERK signaling pathways, thereby reducing expression of downstream key enzymes involved in melanin synthesis. Post hoc correlation analyses further substantiated the integrative link between the downregulation of the melanogenic axis (MITF) and the amelioration of cutaneous oxidative status in programmed offspring. Additionally, EBN suppressed the expression of PMEL and RAB27A, proteins involved in melanosome transport, thereby reducing melanin transfer to keratinocytes. Regarding antioxidant capacity, EBN significantly enhanced the body’s antioxidant state, manifested by increased CAT activity and GCS expression, accompanied by decreased levels of MDA, a lipid peroxidation product. Crucially, these phenotypic and molecular changes were evident at birth (P0) and persisted through weaning (P21), strongly supporting the interpretation that prenatal EBN exposure induced a sustained, developmentally programmed effect on offspring skin, extending beyond a transient pharmacological action. This aligns with the core tenets of the Developmental Origins of Health and Disease (DOHaD) framework [38,45,46,47,48].

Our findings support and extend previous research showing that EBN and its derived peptides exert depigmenting and photo-protective actions in non-pregnant models [34,35]. For instance, in in vitro and in vivo screening models, such as B16 melanoma cells and zebrafish, EBN protein hydrolysates have been shown to reduce melanin content and tyrosinase activity while downregulating the expression of key melanogenic genes, including MITF, TYR, TRP1, and TRP2 [9,49,50]. Standardized EBN extracts protect UVB-irradiated hairless mouse skin and improve human facial wrinkles and skin brightness [7,19]. The present study therefore reveals a novel translation of these actions into a developmental context. Specifically, we demonstrate that when EBN is consumed maternally during gestation, similar anti-elanogenic and antioxidant signatures appear in offspring skin, even in the absence of direct postnatal EBN supplementation. To further strengthen the integrative link between oxidative stress modulation and melanogenesis inhibition, we performed correlation analyses on our endpoint data. The results revealed a strong positive correlation between MITF protein levels and total melanin content, and significant associations between antioxidant markers (CAT, MDA) and pigmentation indices, quantitatively supporting the coordinated downregulation of these pathways. This supports and broadens the concept that EBN acts as a multi-target nutraceutical, capable of simultaneously modulating melanogenesis, melanosome dynamics, and antioxidant pathways through a trans-generational, developmental programming mechanism [7].

This study, utilizing a maternal nutrition intervention model, revealed that edible bird’s nest (EBN) effectively downregulates the expression of the core transcription factor MITF and its downstream melanin synthesis enzymes (TYR, TRP1, TRP2) by synergistically inhibiting the phosphorylation of ERK and CREB in offspring skin. This leads to a significant reduction in skin melanin content. This finding reveals that bird’s nest exerts comprehensive regulation over melanin synthesis—from upstream signaling to downstream products—through a dual-pathway inhibition mechanism. While classical literature suggests that sustained ERK activation may inhibit melanin by degrading MITF [29,30], seemingly contradicting the observed decrease in ERK phosphorylation in this study, this precisely highlights the complexity of ERK signaling regulation. This discrepancy likely stems from ERK’s “biphasic regulation” of melanin synthesis: highly active ERK under specific stimuli negatively regulates MITF degradation, whereas basal ERK activity is crucial for maintaining MITF’s steady-state expression [51,52]. In this study, maternal bird’s nest intervention systematically suppressed basal ERK activity below the threshold required to maintain MITF expression. This finding aligns with reports that various ERK inhibitors reduce melanin production [51,52], collectively indicating that inhibiting rather than activating the ERK pathway achieves skin brightening effects.

Sialic acid is an important active ingredient in bird’s nest. Group SA demonstrated certain brightening effects and melanin synthesis inhibition, consistent with literature reports indicating that sialic acid (Neu5Ac) promotes tyrosinase autophagy degradation and influences melanosome maturation [53,54]. Notably, our study confirms that while isolated sialic acid (Neu5Ac), a key bioactive component in EBN, exerts anti-melanogenic effects consistent with its reported role in promoting tyrosinase autophagy, its efficacy at an equivalent dose was significantly weaker than that of whole EBN. This highlights that EBN’s superior bioactivity stems from its complex matrix—comprising peptides, glycoproteins, and antioxidants—acting synergistically rather than from sialic acid alone [53]. Several factors may contribute to this discrepancy. First, sialic acid in EBN primarily exists as terminal residues on glycoproteins, which may differ in bioavailability, cellular uptake, and signaling compared to the free monomeric Neu5Ac supplemented herein [54]. Second, interspecies differences in sialic acid biology (e.g., endogenous Neu5Gc production in rodents versus humans) may influence receptor engagement and metabolism, cautioning against direct mechanistic extrapolation between models [55]. Thus, our findings underscore that whole food matrices offer integrated physiological regulation—particularly relevant in developmental programming—that surpasses isolated components, supporting the functional value of EBN beyond its individual constituents. This aligns with the growing recognition of the biological advantages of intact food over isolated compounds in modulating complex phenotypic outcomes.

Collectively, our findings delineate a hierarchical model through which maternal EBN intake programs offspring skin pigmentation (Graphical Abstract). At the upstream level, EBN suppresses the melanocortin signaling axis (reduced MC1R and POMC expression) and concurrently inhibits the phosphorylation/activation of two key parallel pathways—CREB and ERK—that converge on the central regulator MITF. This multi-pronged inhibition at the signaling level leads to the sustained downregulation of MITF at the transcriptional and protein levels. At the core transcriptional level, the reduced MITF activity results in the decreased expression of its key downstream target genes encoding the melanogenic enzymes TYR, TRP1, and TRP2, thereby suppressing melanin synthesis. Finally, at the downstream cellular level, EBN also attenuates melanosome maturation (reduced PMEL/gp100) and transfer to keratinocytes (reduced RAB27A), reducing pigment delivery. Concurrently, EBN enhances the skin’s antioxidant capacity (increased CAT/GCS, decreased MDA), creating a tissue microenvironment less conducive to melanogenesis. This coordinated action across multiple hierarchical tiers explains the potent and sustained skin-lightening phenotype. Furthermore, we observed that EBN-induced changes in pigmentation and antioxidant pathways were evident at birth and persisted throughout weaning, indicating a genuine developmental program rather than an acute pharmacological effect. The persistence of these changes from birth through weaning supports their characterization as a developmentally programmed effect. While the precise molecular mechanisms remain to be fully elucidated, the durability of the phenotype invites speculation about potential underlying processes. One plausible, yet currently putative, mechanism involves epigenetic reprogramming [22,28]. For instance, future studies could investigate whether maternal EBN exposure leads to stable epigenetic modifications (e.g., DNA methylation, histone acetylation) at regulatory regions of key pigment genes such as MITF, TYR, and Rab27A. Other speculative avenues include alterations in the specification of neural crest-derived melanocyte progenitors or a sustained re-setting of redox-sensitive signaling pathways. The convergence of EBN’s effects on melanogenesis, antioxidant defense, and melanosome transport is consistent with a model of developmental reprogramming, aligning with the known sensitivity of the melanocyte lineage to early-life environmental cues [40]. However, these hypotheses remain to be tested. As noted above, putative mechanisms such as epigenetic modifications, alterations in skin stem cell populations, or sustained changes in endocrine/immune signaling represent important avenues for future research. Direct confirmation of these mechanisms will require targeted epigenomic, transcriptomic, and lineage-tracing analyses.

Beyond its role in modulating pigmentation, the enhanced antioxidant capacity observed in offspring skin holds broader implications for cutaneous biology and long-term skin health. The upregulation of key enzymes like catalase (CAT) and glutamate–cysteine ligase (GCS), coupled with reduced lipid peroxidation (MDA), indicates a fundamental improvement in the skin’s redox buffering system [38]. This reprogrammed state may confer increased resilience against ubiquitous environmental stressors such as ultraviolet radiation, air pollution, and intrinsic metabolic oxidative load. A robust antioxidant defense is critical for maintaining skin barrier integrity, limiting inflammatory responses, and protecting against oxidative damage to cellular macromolecules like DNA, proteins, and lipids [48]. Consequently, maternal EBN-induced priming of the cutaneous antioxidant network could potentially influence not only pigmentary outcomes but also the skin’s susceptibility to photoaging, inflammatory dermatoses, and other oxidative stress-related pathologies later in life [41]. This perspective aligns with the Developmental Origins of Health and Disease (DOHaD) concept, suggesting that early-life nutritional interventions can shape lifelong tissue resilience [47,56].

However, this study still has certain limitations. First, our findings are derived from a rodent model. Although the core melanogenic signaling pathways (e.g., MITF axis) are evolutionarily conserved, there are critical species-specific anatomical differences. In rodents, the primary pigmentary unit is the hair follicle, whereas in humans, epidermal melanocytes drive constitutive pigmentation [57]. Differences in hair cycle dynamics and the epidermal-melanin unit anatomy may affect the translation of specific mechanisms, such as melanosome transfer [58]. Consequently, while our work provides a robust proof-of-concept for maternal nutritional programming of skin phenotype, direct validation in human-relevant models or clinical studies is needed to confirm translational relevance and safety. Second, we did not perform cross-fostering, and maternal supplementation ceased at delivery. Thus, while the significant effects observed at birth (P0) strongly support a dominant role for in utero programming, potential contributions from postnatal alterations in maternal physiology or colostrum composition cannot be fully excluded. Future studies employing a cross-fostering design would help disentangle prenatal from immediate postnatal effects. Third, our study focused on the main effect of maternal nutrition during gestation and early postnatal development. Melanogenesis and antioxidant responses can be modulated by sex hormones, particularly during later developmental stages such as adolescence. Future investigations should examine the long-term persistence of these effects in mature models and determine whether they exhibit sexual dimorphism [59,60]. Finally, the precise molecular mechanisms underpinning this developmental programming remain to be fully elucidated. The putative involvement of epigenetic modifications or other stable programming processes in mediating these transgenerational effects represents a compelling avenue for future research [61].

Despite these limitations, our results suggest that EBN has potential as a maternal functional food to modulate offspring skin pigmentation by acting on multiple pigmentary and oxidative pathways. This may be particularly relevant in populations where EBN is traditionally consumed during pregnancy and where hyperpigmentation is a cosmetic concern. Carefully designed human studies will be needed to determine safety, optimal dosing, and clinical relevance.

## 5. Conclusions

This study provides evidence that maternal consumption of edible bird’s nest (EBN) during pregnancy can durably program the skin phenotype of offspring, without requiring direct postnatal exposure. The programmed effects characterized by enhanced antioxidant defenses and suppressed melanogenesis are evident at birth and persist throughout early postnatal development (through at least P21). Mechanistically, EBN exerts a multi-pronged, coordinated action by simultaneously downregulating the melanogenic axis (via the MITF–TYR/TRP–1 pathway), attenuating melanosome transport, and upregulating key antioxidant enzymes. Notably, the whole EBN matrix exhibited superior efficacy compared to an equivalent dose of its primary constituent, sialic acid, highlighting the functional importance of the intact food matrix and synergistic interactions among its components. Collectively, these findings position EBN as a promising maternal functional nutrient that programs offspring skin health beyond mere pigmentation modulation. They offer a novel perspective on how complex maternal diets can influence developmental trajectories and suggest early-life nutritional strategies for promoting cutaneous resilience and long-term skin health. As a proof-of-concept established in a rodent model, these results warrant future validation in human studies to confirm translational relevance, safety, and efficacy.

## Data Availability

The data presented in this study are available within the article. The original data are not publicly available due to being part of an ongoing enterprise collaboration project; however, they are available from the corresponding authors upon reasonable request.

## Supplementary Materials

The following supporting information can be downloaded at: https://www.mdpi.com/article/10.3390/nu18071083/s1, Table S1: Primers used for quantitative real-time PCR (qRT-PCR) analysis of melanogenesis-related genes.
