# Supplementary material for: Prenatal Edible Bird’s Nest Supplementation Attenuates Offspring Skin Pigmentation via Dual Inhibition of CREB and ERK Signaling to Downregulate MITF-TYR Axis
Source: Nutrients. 2026 Mar 28;18(7):1083. doi: 10.3390/nu18071083 (PMC13074263; doi:10.3390/nu18071083)
Supplement: Supplementary file 1 [file nutrients-18-01083-s001.zip › nutrients-4123550-supplementary.pdf]

Supplementary Table S1 - Primers used for quantitative real-time PCR (qRT-PCR) analysis of melanogenesis-related genes

This table lists the forward and reverse primer sequences for the target genes TYR, TRP1, TRP2, MITF, MC1R, and POMC, along with the corresponding amplicon sizes. All primers were designed using (Primer-BLAST, https://www.ncbi.nlm.nih.gov/tools/primer-blast/) and synthesized by (Beijing Tianyi

Gene	Primer Direction	Sequence (5’ -> 3’)	Length
TYR	Forward	GCTTCCTCTACCTGGACAAG	20
	Reverse	TTGTCGTTGGTGAGCATCCA	20
TRP1	Forward	CCGAAACACAGTGGAAGGTT	20
	Reverse	TCTGTGAAGGTGTGCAGGAG	20
TRP2	Forward	GCAAGAGATATACCCAGAGG	20
	Reverse	AGGTCCAGTAGGGATCATAC	20
MITF	Forward	TACAGCAACCAGAGCCTTTG	20
	Reverse	CCTCTTTTTCACAGTTGGAG	20
MC1R	Forward	GTACCACAGCATCGTGACCTT	21
	Reverse	AGGAAGCAGAGGCTGGACA	19
POMC	Forward	CCTCCTGCTTCAGACCTCCA	20
	Reverse	GCGTTCTTGATGATGGCGTT	20

Huiyuan Biotechnology Co., Ltd. Beijing, China)
